# p-adic numbers encode complex networks

**DOI:** 10.1038/s41598-020-79507-4

**Published:** 2021-01-08

**Authors:** Hao Hua, Ludger Hovestadt

**Affiliations:** 1 School of Architecture, Southeast University, 2 Sipailou, Nanjing, 210096 China; 2 Key Laboratory of Urban and Architectural Heritage Conservation (Southeast University), Ministry of Education, Nanjing, China; 3 Department of Architecture, ETH, Zürich, Switzerland

**Keywords:** Mathematics and computing, Physics

## Abstract

The Erdős-Rényi (ER) random graph *G*(*n*, *p*) analytically characterizes the behaviors in complex networks. However, attempts to fit real-world observations need more sophisticated structures (e.g., multilayer networks), rules (e.g., Achlioptas processes), and projections onto geometric, social, or geographic spaces. The p-adic number system offers a natural representation of hierarchical organization of complex networks. The p-adic random graph interprets *n* as the cardinality of a set of p-adic numbers. Constructing a vast space of hierarchical structures is equivalent for combining number sequences. Although the giant component is vital in dynamic evolution of networks, the structure of multiple big components is also essential. Fitting the sizes of the few largest components to empirical data was rarely demonstrated. The p-adic ultrametric enables the ER model to simulate multiple big components from the observations of genetic interaction networks, social networks, and epidemics. Community structures lead to multimodal distributions of the big component sizes in networks, which have important implications in intervention of spreading processes.

## Introduction

Number theory has a reputation of “unreasonable effectiveness.” Perhaps the most famous example is the Fibonacci numbers, which are closely related to golden ratio, plant growth (Phyllotaxis), and DNA patterns. Over the past years, a few developments have begun to unfold the potentials of number theory in understanding complex networks^1,2^. This work employs the p-adic numbers for modeling complex networks based on the Erdős-Rényi (ER) random graph. The p-adic number system gives an extension of the ordinary arithmetic of rational numbers (${\mathbb {Q}}$) in a way distinct from the common extension of ${\mathbb {Q}}$ to real numbers (${\mathbb {R}}$) and complex numbers (${\mathbb {C}}$). The p-adic number system can be applied in various scientific fields. One groundbreaking application in physics is the p-adic AdS/CFT^3^. Khrennikov et al.^4^ developed p-adic wavelet for modeling reaction-diffusion dynamics. Applications in biology include the models for hierarchical structures of protein^5^ and genetic code^6^.

### Networks, behaviors, and health

There is growing interest on a unified theory of complex networks from various fields^7–9^. The formalism commenced with graph theory in mathematics. The emergence of giant component is essential for the evaluation of random graphs^10–12^. Network analysis revealed hidden structures in social and economic systems^13,14^. Exponential random graph^15^ and stochastic blockmodels^16,17^ were developed. Many statistical mechanics in physics, e.g., percolation^18^ and time series^19^, are modeled with complex networks. Network models are closely associated with epidemiology^20,21^ and public health^22^. Chains of affection^23^ is a classical work revealing the network structure as a critical factor in public health. The spatiotemporal dynamics^20^ is essential for understanding the epidemic processes.

The complex networks also play an essential role in the microscopic scale. Numerous researches in biology rely on the curation and archival storage of protein, genetic, and chemical interactions for all major model organism species and humans (e.g. BioGRID and STRING database). The disease network^24,25^, protein-protein interaction network, and gene network^26^ contributed to the network approaches to life science^27^. In the near future, the network advances in biology, such as drug targeting^28,29^ and network medicine^30^, might be critical for improving our health.

### Erdős–Rényi model and extensions

The classical ER model has inspired the continuous development of a rich spectrum of sophisticated models. There are three major approaches:

- Creating more complex structures such as multilayer networks^31,32^ and multiplex networks^33,34^. Complex networks often exhibit community or module structures^7^.
- Manipulating the rules for constructing edges. The Achlioptas process picks two candidate edges each time^18^ for competitive graph-evolution. ER process, Bohman and Frieze process, and product rule process were compared with one another for analyzing explosive percolation^35^.
- Projecting graphs onto social, geometric, or geographic structures. Major developments in that aspect include the hyperbolic networks^36^, spatial preferential attachment^37^, inhomogeneous random graph^38,39^, and spatial networks^30^.

### Findings

Common attempts to modeling the hierarchical structures^7^ are reflected by various notions including subpopulation, subgraph, mixing pattern, community, and module. We postulate that hierarchical structures are naturally encrypted in a standard graph. Consequently, imposing additional structures onto a graph to enrich its behavior is not always necessary. The key is the p-adic absolute value^40^. According to Ostrowski’s theorem, every non-trivial absolute value on ${\mathbb {Q}}$ is either the usual real absolute value or the p-adic absolute value. Various hierarchical structures can be represented by p-adic integers as nodal indices. The network topology and relative strengths between connections are unified as p-adic distances between numbers.

The p-adic random graph (PARG), probably the simplest model of inhomogeneous networks, offers a flexible method to simulate various observations in complex networks, especially the phenomenon of multiple big components. Degree distribution is a key property for distinguishing random, free-scale, and small world networks. However, PARG indicates that two random graphs with identical degree distribution may produce significantly different component sizes.

We fit PARG to the component size distribution of the genetic interaction networks, and also to the joint distributions of big components in COVID-19 outbreaks. The experiments imply that the community structures are responsible for the multimodal distributions of the sizes of big components. The largest or the second largest component could be more stable at (multiple) specific sizes. Therefore, maintaining a local peak could be valuable for intervening the spreading processes.

## p-adic random graph

**Figure 1:**
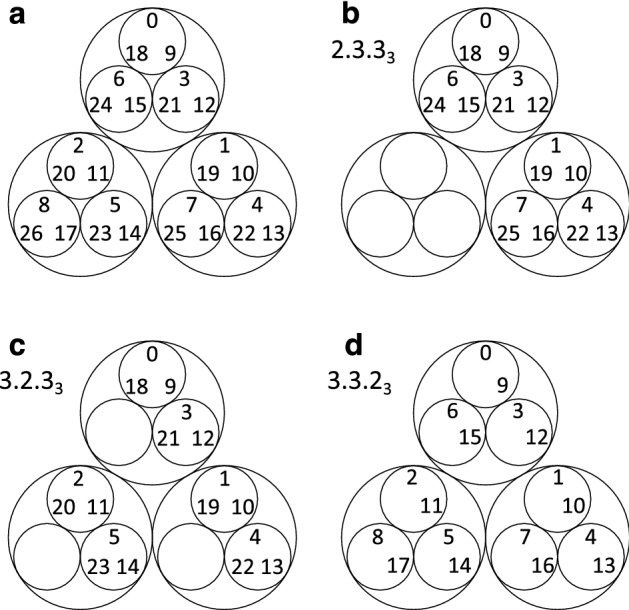
Hierarchical structures of natural numbers. (**a**) Circular tree-map for the hierarchical structure of 3-adic numbers. (**b–d**) The digit notations specify the hierarchical arrangements.

In contrast to the celebrated ER network, another early prototype of random graph, the Rado graph, has been rarely revisited. The Rado graph employs the binary number system to encode the graph edges, using Ackermann coding of hereditarily finite sets. PARG explores the fundamental nature of integers to encode the probability of connecting a pair of nodes. The p-adic number system extends the ordinary arithmetic of rational numbers^3,40^. Our PARG model will focus on the p-adic metric on nonnegative integers. An integer’s *r*-adic (picking a prime number *r*) absolute value is the reciprocal of the largest power of *r* that divides it. For example, $|40|_2=1/8$ (let *r*=2, then the 2-adic absolute value of 40 equals 1/8), $|40|_3=1$, $|40|_5=1/5$. Such absolute value is the most significant example of ultrametrics^40^. Each node is naturally associated with an integer, i.e., its index in the ordered set of nodes, or an arbitrary (unique) integer can be assigned to each node. The probability of connecting any pair of nodes *i*, *j* is proportional to the p-adic closeness between the two nodal indices $v_i, v_j$ :

1 $$\begin{aligned} p_{ij}= \frac{p^*}{ |v_j -v_i|_r} \end{aligned}$$

for $i,j\in [1,n],\; i<j$ (assuming $i<j \Leftrightarrow v_i<v_j$). $p^*$ is a constant as the probability in the ER sense. When comparing ER and PARG, we normalize the PARG probability so that $\sum _{ij} p_{ij} =n(n-1) p^*/2$. As a result, the number of edges in ER equals that in PARG. The p-adic distances encode a hierarchical structure, as shown in the circular tree-map in Fig. 1a. The distances between any pair of numbers from the same small circle and the same big circle are 1/9 and 1/3, respectively. If the two numbers are from different big circles, their distance is 1.

The digit format of a p-adic number is intuitive, for example, $201_3=2\cdot 3^0 +0\cdot 3^1+1\cdot 3^2=11$. One can construct a set of integers (as the nodal indices) in their digit formats by

2 $$\begin{aligned} u_0 . u_1 . \cdots .u_m \;_r\, {\mathop {=}\limits ^{ \text {def}}}\, \{ a_0 a_1 \cdots a_m \;_r \; | \; 0\le a_i < u_i \text { for } i=0,1,\cdots ,m \} \end{aligned}$$

where *r* is the chosen prime number. We call the model a full PARG if $u_0=u_1=\cdots = u_m=r$ (i.e., $n= r^{m+1}$), otherwise, we call it a regular PARG (i.e., $n= \prod _i u_i$). The nodal indices can also be arbitrary digits under a given prime *r*, which leads to a general PARG. The expression (2) facilities the enumeration of hierarchical structures. For example, $3.2.3_3$ fully describes a hierarchical structure as shown in Fig. 1c. More examples are in illustrated in Fig. 1. A notation such as $G(3.2.3_3, p^*)$ fully specifies a PARG.

**Figure 2:**
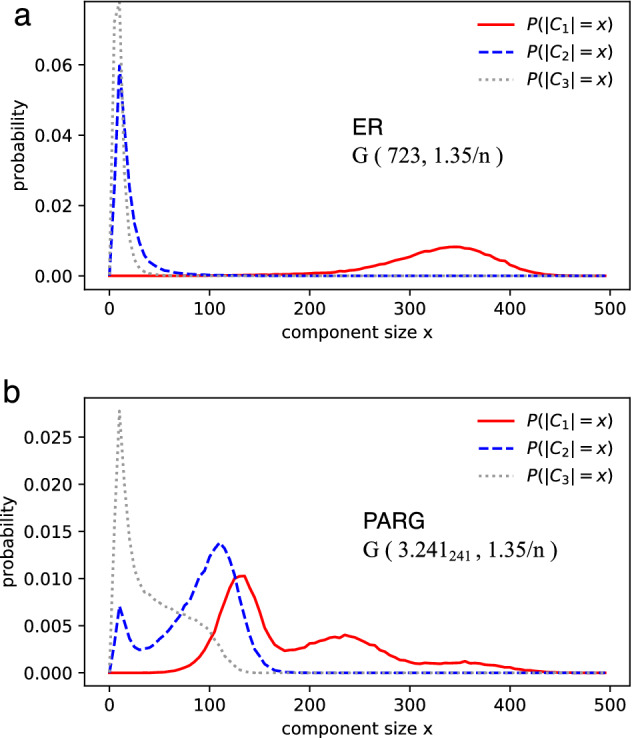
Size distributions of big components in (**a**) ER and (**b**) PARG. The probabilities are drawn from 60,000 of random realizations (see “Methods” section). Both models have the same degree distribution, and they contain the same number of edges (487 edges), as PARG’s connecting probability (1) is normalized by $\sum _{ij}p_{ij}= n(n-1) p^*/2$.

PARG implements a Bernoulli process on all pairs from a set of p-adic numbers. Let $p_k$ represent the probability of a randomly chosen node with degree *k*. In the ER model, $p_k$ follows a Binomial distribution. It becomes a Poisson distribution in the limit of large *n*. By contrast, *n* in PARG equals the number of individuals in observations. Because of the symmetric connectivity in regular PARG, $p_k$ can be obtained from the degree distribution of one node:

3 $$\begin{aligned} p_k= \sum _{\alpha _0,\alpha _1,\cdots ,\alpha _m} \prod _{i=0}^m (r^i p^*)^{\alpha _i} (1-r^i p^*)^{d_i-\alpha _i} \left( {\begin{array}{c}d_i\\ \alpha _i\end{array}}\right) \end{aligned}$$

where $\sum _{i=0}^m d_i =n-1$ holds. $d_i$ denotes the number of links (from the chosen node) with probability $r^ip^*$ according to (1-2). The numbers $\alpha _0,\alpha _1,\cdots ,\alpha _m$ denote all combinations satisfying $k=\sum _{i=0}^m \alpha _i$. Numeric computing of (3) indicates that $p_k$ in PARG and that in ER are almost identical. The two models can have the same degree distribution and the same number of edges. However, there could be significant differences in the size distributions of big components in PARG and ER (Fig. 2, Supplementary Note 1, Table S1 and S2). The largest components in PARG may exhibit multimodal distribution due to the hierarchical structure. This implies that certain sizes of (multiple) big components are more statistically stable than other sizes.

## Sizes of big components

The relative sizes of multiple big components in complex networks were much less studied compared to the studies carried out on the giant component^12,41^. Analytical methods^11,42^ and generating functions^10^ have been widely employed for analyzing the component sizes. Rather than let $n\rightarrow \infty$, *n* in PARG is equal to the number of relevant individuals in observations. As a result, the sizes of simulated components are similar to that in ground truth. When *n* is finite, numeric random realizations are suited to evaluate the various probabilities about component sizes (see “Methods” section).

When *n* is fixed, fitting ER model to empirical data only involves the single parameter *p*, while PARGs involve two kinds of parameters: the probability *p* and the hierarchical structures represented by (2). The configuration space of (2) is very vast, even when $n<1000$, so we opted for ad hoc heuristics to choose the hierarchical structures that fit relatively well to observations. The heuristics includes: (1) scaling the distances. For example, $7.5.6_r$ with $r=7, 11, 13,\cdots$ represent the same hierarchical structure, though the distances between the levels are scaled. (2) Flat vs. deep hierarchy. For instance, both $16.16_{17}$ and $2.2.2.2.2.2.2.2_2$ refer to 256 nodes. The former is made of 16 groups (each contains 16 nodes), while the latter’s structure looks like a high tree.

Based on the hierarchical structures in PARG, the following experiments analyze multiple big components, especially $5|C_2|>|C_1|$, as static structures observed in networks. The topics range from microscopic networks, such as biological networks^26,28^, to macroscopic networks, such as epidemics^21,43^.

### Genetic interaction networks

**Figure 3:**
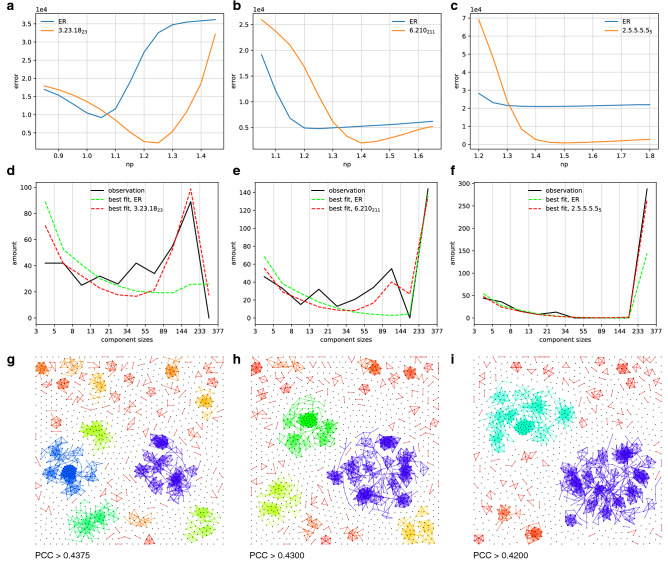
Simulating the sizes of big components in genetic interaction network. (**a–c**) The errors (4) of the simulations of component sizes from ER and PARG with distinct values of *np*. The errors from 2,000 realizations are averaged to obtain relatively accurate evaluation. (**d–f**) The component size distributions of the best fit from ER and PARG, averaged over 2,000 random realizations. The *y*-axis (amount) is defined as $(b_{i+1}-b_i) \theta _i$ in consistent with error function in (4). (**g–i**) The components in genetic interaction network of yeast genes (theCellmap.org). The PPC thresholds for connecting a pair of nodes are 0.4375, 0.43, and 0.42, respectively. The color (hue) of edges indicate the size of the components (largest: blue, smallest: red).

The essential role of genetic interaction networks plays in biology has been lately revealed^25^. The essential genetic interaction network of yeast genes (theCellmap.org) contains 1,261 mutant strains. Their interactions have been characterized by Pearson correlation coefficient (PCC). Genes with highly correlated genetic interaction profiles (PCC>0.4) form clusters of specific pathways or protein complexes^26^. We set three PCC thresholds above 0.4 to obtain three graphs with distinct big components, as shown in Fig. 3g–i. We count the components sizes falling into the predefined intervals of Fibonacci numbers ($b_{i+1}=b_i+b_{i-1}, b_0=3$). Let $\theta _i$ be the number of simulated components whose sizes fall between $b_i$ and $b_{i+1}$, and $\theta _i^*$ be that in ground truth. The error of a random realization is given by

4 $$\begin{aligned} \sum _i \left[ (b_{i+1}-b_i) (\theta _i - \theta _i^*) \right] ^2 \end{aligned}$$

The averaged error from many random realizations yields a relatively accurate evaluation. ER and PARG with distinct values of *np* lead to different errors (Fig. 3a–c). Equipped with (configurable) hierarchical structures, PARGs fit better to observations than ER. The component size distributions of the best fits are shown in Fig. 3d−f.

### Protein-protein interaction

Exploring the protein interaction networks of proteins poses a major challenge in biomedicine. Protein-protein interaction (PPI) is crucial to understanding cellular pathways and human diseases^44^. The following experiment creates a graph from a set of 408 S. cerevisiae protein complexes as^45^. The graph nodes represent individual proteins from these complexes. An edge is constructed between two nodes (proteins) if they belong to the same complex. The graph includes 1,628 nodes and 11,249 edges.

**Figure 4:**
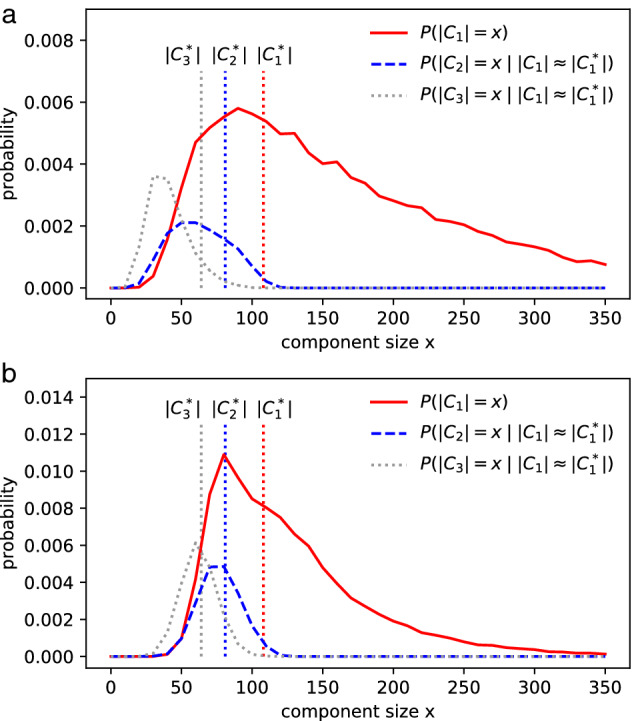
The distribution of the first 3 largest components in ER and PARG. $n=1628$ in the PPI network. (**a**) The best fit of ER model, $S_{tri}$=0.112 at *p*=1.035/n. (**b**) The best fit of $G(11.148_{521}, 1.225/n)$, $S_{tri}$=0.307 when $p^*$ =1.20/n. The ground truth ($|C_1^*|$=108, $|C_2^*|$=81, $|C_3^*|$=64) in PPI network of S. cerevisiae is marked with vertical dotted lines. The details of realizations and definitions can be found in “Methods” section.

We define the similar metric $S_{duo}$ and $S_{tri}$ (see “Methods” section) to compare the simulated component sizes with the ground truth. PARG fits better ($S_{tri}$=0.307) than the ER model ($S_{tri}$=0.112) to the PPI network, as shown in Fig. 4. The simulation data can be found in supplementary Table S3 and S4. It means that the chosen PARG has a higher probability that the sizes of its big components resemble those in the PPI network.

### Instrumental resource street network

There has been growing attention to the impact of social networks on health^46^. For example, homeless youth is an active research field, including analysis and interventions. Social networks of homeless youth^47,48^ are vital for understanding and intervening the observed phenomena.

This experiment involves a social network about employment services utilization among homeless youth. The original research^49^ queried 136 homeless youth in Los Angeles in 2008. Four distinct networks were constructed from the same population, according to instrumental, emotional, employment services use, and sociometric relationship respectively. Only the instrumental network ($|C_1^*|$=30, $|C_2^*|$=13) satisfies $5|C_2^*|>|C_1^*|$, i.e., the second largest component is large enough. Regarding the similarity metric $S_{duo}$, the ER model attains the maximum similarity ($S_{duo}$=0.155) at $p=1.08/n$; while $G(10.14_{353}, 1.75/n )$ has a much higher similarity ($S_{duo}$ =0.316). The community structure in the PARG corresponds to the social or geographical networks of the homeless youth; although the map between the two is still elusive.

We also fit the hyperbolic networks^36^ and the Achlioptas process^35^ to the observed component sizes ($|C_1^*|$=30, $|C_2^*|$=13). Details can be found in “Methods” section. The random hyperbolic graph^50,51^ reaches the maximum similarity ($S_{duo}$=0.266) when $C=-9.3$, $\alpha =10$, $D=0.0684R$. The Achlioptas process with product rule (PR) has the maximum similarity ($S_{duo}$=0.221) when the number of edges is equal to 100. So PARG outperforms the other two models in this case.

### Spreading of coronavirus

Coronavirus^52^ has spread among many Chinese cities since the end of January 2020. The incubation period^53^ and possible mild symptoms^54^ made the prevention more complicated. Social networking sites (or local officials) reported traces of infected people. Relationships between the infected (and those who had close contacts with them) were also investigated. From a point of view of networks, the cities exhibited three distinct patterns: 1) No big components. Shenzen reported 416 confirmed cases by February 20, 2020. The largest cluster has only 9 people. 2) A giant component. Xinyu reported 110 confirmed cases by February 10, 2020. The giant component consists of 52 cases, nearly half of the infected population. The second largest component has only six cases. 3) Multiple big components. Tianjin reported 136 confirmed cases by February 3, 2020. The first, second, and third largest clusters contain 44, 17 and 11 cases, respectively, which are related to a huge department store, the railway, and a residential area, respectively.

**Figure 5:**
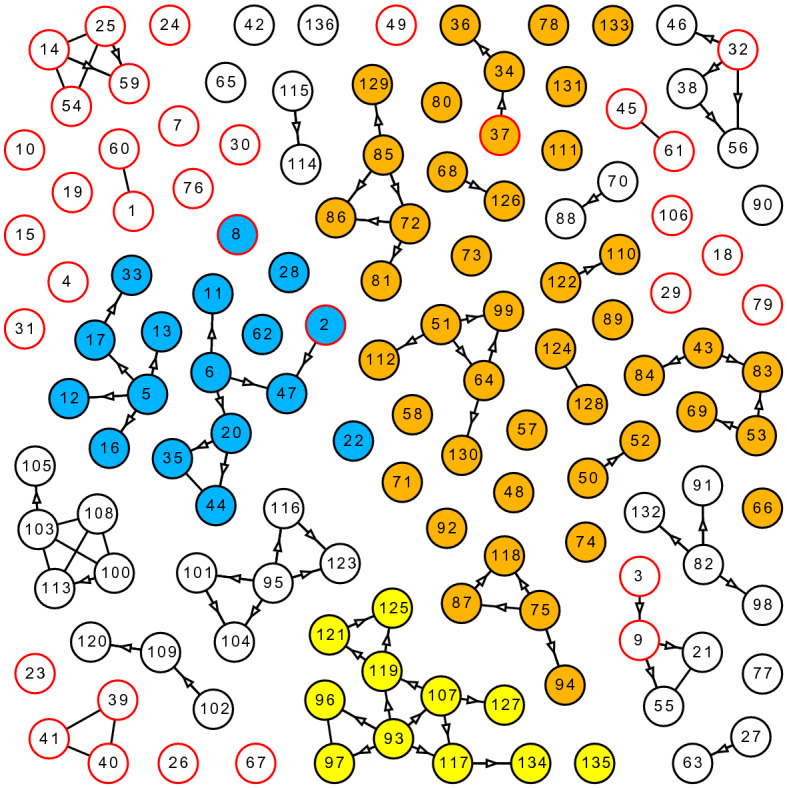
Network of infected cases of COVID-19 in Tianjin. A red outline indicates that the case was infected outside Tianjin; a black outline indicates that the case was infected inside Tianjin. An edge refers to the close contact between the two nodes (persons). An arrow indicates the infection process between the two nodes. The 44 nodes in orange represent the infected people who either worked or visited the department store. The 17 blue nodes are related to the railway. The 11 yellow nodes represent the cases from a residential area. Later investigation indicates that the orange and yellow nodes are related, therefore they form a larger component of 55 nodes.

**Figure 6:**
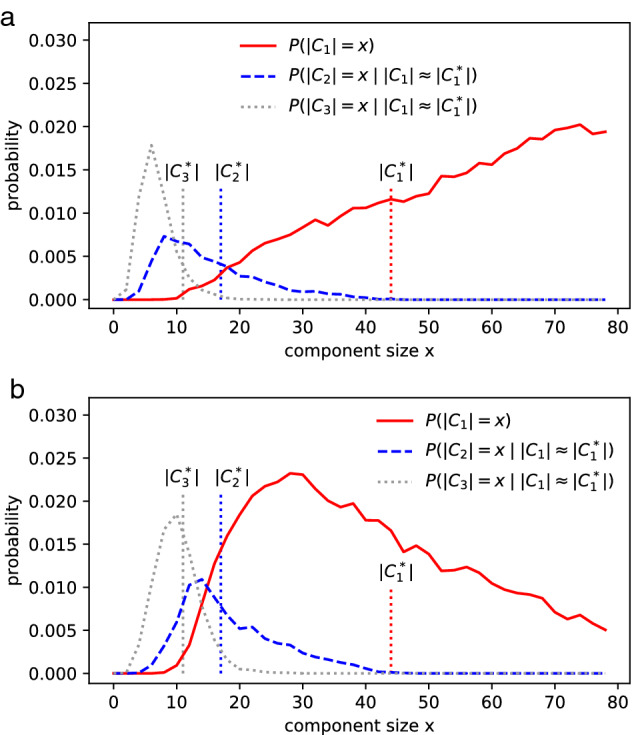
Fitting ER and PARG to the observed cluster sizes ($|C_1^*|$=44, $|C_2^*|$=17, $|C_3^*|$=11) in Tianjin. Both models contain 136 nodes and run 20,000 random realizations. (**a**) ER model has 93.1 edges. It reaches the maximum similarity ($T_{tri}$=0.0024) when $p=1.379/n$. (**b**) $G(8.17_{59}, 1.49/n)$ consists of 100.6 edges. The PARG has a much higher similarity $T_{tri}$=0.00916.

We focus on Tianjin’s infection network (Supplementary Note 1, Table 5), which consists of multiple big components. A graph is created to visualize the relationship between the infected when the outbreak was around its peak, as shown in Fig. 5. The simulation data can be found in Supplementary Table S6 and S7. The similarity metric $S_{tri}$ could be biased when $|C_3^*|$ is quite smaller than $|C_1^*|$, so the similarity metric $T_{tri}$ (see “Methods” section) is employed in this case to fit the models to the observed clusters, as shown in Fig. 6. The PARG $G(8.17_{59}, 1.49/n)$ has the highest similarity $T_{tri}$=0.00916.

We also compared the results of the random hyperbolic graph and the Achlioptas process with that of PARG. The random hyperbolic graph reaches the maximum similarity ($T_{tri}$=0.0150) when $C=-9.65$, $\alpha =1$, $D=0.04294R$. The Achlioptas process with Bohman Frieze (BF) rule has the maximum similarity ($T_{tri}$=0.00825) when the number of edges is equal to 93. So the random hyperbolic graph fits best to this case.

**Figure 7:**
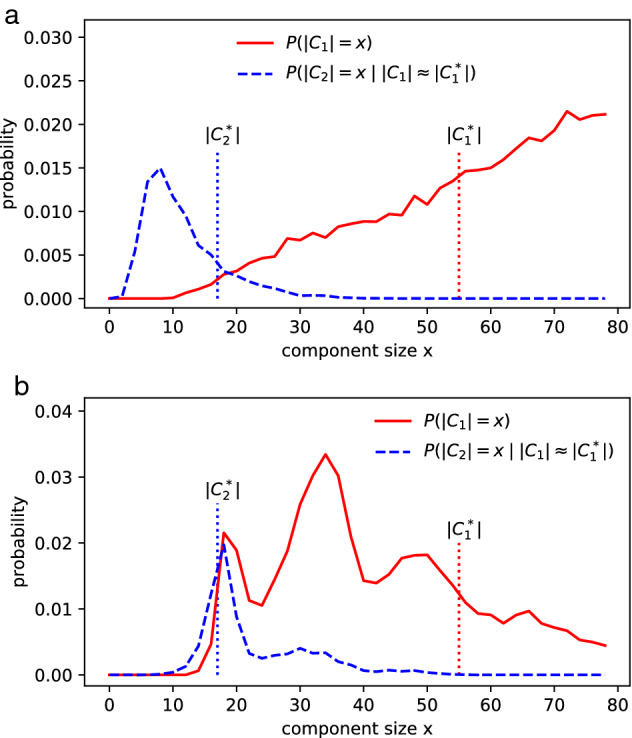
Fitting ER and PARG to the observations ($|C_1^*|$=55, $|C_2^*|$=17) in Tianjin. Both models contain 136 nodes and run 20,000 random realizations. (**a**), ER model contains 95.2 edges. It reaches the maximum similarity ($T_{duo}$=0.0161) when $p=1.41/n$. (**b**), $G(7.20_{179}, 1.90/n)$ contains 128.2 edges and has a much higher similarity $T_{duo}$=0.0498.

A later investigation indicates that the 11 cases (in yellow, Fig. 5) are probably related to the department store as well. In this new perspective, the two big clusters form the largest component of 55 nodes. We employ the metric $T_{duo}$ to fit modes to this new observation ($|C_1^*|$=55, $|C_2^*|$=17), as shown in Fig 7. The simulation data can be found in Supplementary Table S8 and S9. The great variety of hierarchical structures enable PARG to fit relatively well to observations from distinct perspectives.

## Discussion

The size distributions of big components in complex networks are attributed to the structure of physical world; the behaviors of agents (nodes) and the information transmitting between them; and the observer (how to look at the events). The ER model offered prominent findings of component sizes in networks, however, it rarely fits the joint distribution P($|C_1|\approx |C_1^*|$, $|C_2|\approx |C_2^*|$, $\cdots$) in real observations. A successful strategy is introducing inhomogeneous structures or selective rules (for constructing edges) to the random graph to increase its versatility. PARG probably provides the simplest way to fully describe a hierarchical structure in an ER-like model.

PARG interprets the *n* in ER model as the cardinality of a set of natural numbers (nodal indices). Consequently, the probability *p* can be weighted by distances between the nodal indices. In number theory (Ostrowski’s theorem), any non-trivial definition of absolute value on ${\mathbb {Q}}$ is either the conventional one or the p-adic absolute value. So, the p-adic ultrametric reveals the natural hierarchical structures hidden in any graph with indexed nodes. PARG blurs the boundaries between the topology approaches (e.g., multiplex networks) and the geometric approaches (e.g., hyperbolic networks).

In our PARG approach, *n* denotes the number of observed individuals, whereas in previous ER-like models $n\rightarrow \infty$. The limit of *n* facilities analytical approaches^10,11^, while a relatively small finite *n* is convenient for numerical random realizations. Random graph theories such as explosive percolation^35^ and synchronization^55^, deeply revealed the dynamics of the emerged components. By contrast, this work explores the sizes of resultant (static) components from observations or simulations. The results imply that the proportions between the big component sizes are closely associated with the hierarchical structures of complex networks. This implication is in contrast to previous emphasis^10,27,56^ on degree distribution as the fingerprint of network structures.

We fit PARGs and other random graphs to observations from various types of networks. The PARG outperforms the ER model and the Achlioptas process. The random hyperbolic model fits better than PARG to certain cases but worse than PARG to other cases. The simulations of PARG show that the size of big components, e.g., $P(|C_1|=x)$ and $P(|C_2|=x)$, can exhibit multimodal distribution (e.g., Figs. 2b and 7(b)) due to their modular structures. In the case of multimodal distribution, the first peak of $P(|C_2|=x)$ could be very close to the last peak of $P(|C_1|=x)$. Thus, one may distinguish the major mode from the minor modes when investigating the giant component. One present challenge in network epidemic modeling^57^ is designing network-based interventions. Current strategies include targeting high-degree nodes or central nodes. The multimodal distribution of $P(|C_i|=x)$ has implications in controlling the spreading processes in networks. Since $P(|C_i|=x)$ has more than one local peak, it might be possible to predict and maintain the big components’ growth around a local peak.

## Methods

### Random graph implementation

All random graphs are created through random experiments implemented in the Java programming language (Java 1.8 with Eclipse IDE). Given a connecting probability *p* (in the ER context), an edge is included in the graph if

$$\begin{aligned} p > rand \end{aligned}$$

where *rand* stands for a random number between 0 and 1, generated by the method nextDouble() from the java.util.Random class. The method generates a stream of pseudorandom numbers via linear congruential generator (LCG) with modulus $2^{48}$.

A disjoint-set data structure (Union-Find algorithm) is employed to find all components in graph. *N* random realizations of ER or PARG yield an ensemble of binary numbers

$$\begin{aligned} \delta _{ix}^j= {\left\{ \begin{array}{ll} 1, \text { if } |C_i^j|=x \\ 0, \text { otherwise} \\ \end{array}\right. } \end{aligned}$$

for $x=1,2,\cdots , n$. $|C_i^j|$ denotes the size of the *i*th largest component in the *j*th realization. Then one can evaluate the size distribution of the big components by

$$\begin{aligned} P(|C_i|=x) =\frac{1}{N} \sum _{j=1}^N \delta _{ix}^j \end{aligned}$$

One can choose an a prior function to measure the similarity between the simulated components sizes and the observed sizes. For example, Poisson distribution can be used to define the similarity between $|C_i^j|$ and the observation :

$$\begin{aligned} \Psi _i^j= \frac{|C_i^*|!\; |C_i^*|^{\left( |C_i^j|- |C_i^*|\right) } }{|C_i^j|!} \end{aligned}$$

or one can use the normal distribution:

$$\begin{aligned} \Psi _i^j= \exp \left( \frac{ \left( |C_i^j|-|C_i^*|\right) ^2}{s^2 |C_i^*|^2} \log \frac{1}{2} \right) \end{aligned}$$

where *s* is a constant (typically $s=0.1$, so that 10% deviation from the truth results in 1/2 similarity). Simulations in this work employed the later definition. The probability distribution of $|C_2|$ under the condition $|C_1|\approx |C_1^*|$ is given by

$$\begin{aligned} P(|C_2|=x \; \big |\; |C_1| \approx |C_1^*| ) =\frac{1}{N} \sum _{j=1}^N \Psi _1^j \delta _{2x}^j \end{aligned}$$

Likewise, $$P(|C_3|=x \; \big |\; |C_1| \approx |C_1^*| ) =\frac{1}{N} \sum _{j=1}^N \Psi _1^j \delta _{3x}^j$$.

We define the objective function

$$\begin{aligned} S_{duo}= \frac{1}{2N} \sum _{j=1}^N \sum _{i=1}^2 \Psi _i^j,\;\; S_{tri}= \frac{1}{3N} \sum _{j=1}^N \sum _{i=1}^3 \Psi _i^j \end{aligned}$$

for a random graph to measure whether its first two/three largest component sizes are close to that in observation. When $|C_2^*|$ or $|C_3^*|$ is much smaller than $|C_1^*|$, the following metric would be more appropriate:

$$\begin{aligned} T_{duo}= \frac{1}{N} \sum _{j=1}^N \Psi _1^j \Psi _2^j, \;\; T_{tri}= \frac{1}{N} \sum _{j=1}^N \Psi _1^j \Psi _2^j \Psi _3^j \end{aligned}$$

### Genetic interaction network of yeast genes

The data of yeast genes is from https://thecellmap.org/ costanzo2016/. The networks in Fig. 3g–i are drawn from the data of the Essential $\times$ Essential network, which involves 1,261 mutant strains. So, each network in Fig. 3g–i consists of 1261 nodes. The PPC values between the mutant strains are obtained from the genetic interaction profile similarity matrices. An edge is included in the graph if the corresponding PPC value is above the predefined threshold. The graphs are projected onto squares using our Java program, as shown in Fig. 3g–i. In a dynamical process, the agents (nodes) push away from each other, while each edge drags the two end nodes into a fixed range. The color (hue) of edges indicates the size of the relevant component.

### Protein network of yeast genes

The data of S. cerevisiae protein complexes is obtained from the additional File 1 of^45^. We programmed a Java application to read the table, construct the 1,628 nodes (proteins) and 11,249 edges (a pair of nodes belong to a same complex), and find the components via Union-Find algorithm.

### Random hyperbolic graph

Our implementation follows the formulation in^50,51^. $R=2\ln n + C$ denotes the radius of the disc. The probability density for the radial coordinate *r* of a point $(r, \phi )$ is given by

$$\begin{aligned} \alpha \frac{ \sinh (\alpha r)}{ \cosh (\alpha R) -1} \end{aligned}$$

We use inverse transform sampling to generate the radii of the points, i.e., $r= \frac{1}{a} arcosh (1+ \cosh (\alpha R) x -x)$ where $x\in (0,1)$ denotes a random number from the uniform distribution. Our experiments generate *x* by nextDouble() in Java. For each pair of nodes *u*, *v*, a link is added to the graph if $d(u,v) < D$ where $D \in (0,R]$ is a constant and *d*(*u*, *v*) denotes the distance between the two points in the hyperbolic space.

$$\begin{aligned} \cosh ( d(u,v)) = \cosh r_u \cosh r_v - cos(\theta _u - \theta _v) \sinh r_u \sinh r_v \end{aligned}$$

### Achlioptas process

At each time step of the Achlioptas process^18,35^, two edges $e_1$ and $e_2$ compete for addition. Suppose $e_1$ involves two components of size $|C_a|,|C_b|$; $e_2$ involves two components of size $|C_c|,|C_d|$, we consider three types of competing rules:

- add $e_1$ if $|C_a|+|C_b| < |C_c|+|C_d|$, add $e_2$ otherwise.
- product rule (PR): add $e_1$ if $|C_a||C_b| < |C_c||C_d|$, add $e_2$ otherwise.
- Bohman Frieze (BF) rule: add $e_1$ if $|C_a|=|C_b| =1$, add $e_2$ otherwise.

Our experiment treats the number of edges (added to the graph) as the parameter.

## Conclusions

A number theory approach to random graph is proposed. A set of *n* random numbers generates an *n* by *n* adjacency matrix whose binary elements follow the probability (1). Thus, a hierarchical structure is implemented through ultrametrics^40,58^. The simplicity of the digit form (2) for hierarchical structures of random graph facilitates the enumeration of different setting of clusters and hierarchies. In contrast to mapping complex structures or rules from real world to random graph, our PARG approach explores the complex structures in numbers^2,59^ which might be rich enough for modeling complicated observations. An alternative point of view suggests that a plain graph can unfold a hidden hierarchical structure, based on an indispensable definition of absolute value.

The proposed PARG model is more abstract than multilayer networks, multiplex networks, and social-geographical models, but more concrete than ER-like models without community structures. Therefore, a future framework of research would consist of two interconnected levels: (1) Searching for hyper parameters, such as hierarchical structures, in PARG, given empirical data and (2) Constructing the ad hoc realistic models, for example, the biomolecular environments in cell or the social-geographical structures in a city, which the PARG model is projected onto.

## Supplementary Information

Supplementary Information 1.

Supplementary Table S1.

Supplementary Table S2.

Supplementary Table S3.

Supplementary Table S4.

Supplementary Table S5.

Supplementary Table S6.

Supplementary Table S7.

Supplementary Table S8.

Supplementary Table S9.
