# Supplementary material for: p-adic numbers encode complex networks
Source: Sci Rep. 2021 Jan 8;11:17. doi: 10.1038/s41598-020-79507-4 (PMC7794417; doi:10.1038/s41598-020-79507-4)
Supplement: Supplementary file 1 — Supplementary Information 1. [file 41598_2020_79507_MOESM1_ESM.pdf]

Supplementary Information
p-adic Numbers Encode Complex Networks

Hao Hua[1,*], Ludger Hovestadt[2]
School of Architecture, Southeast University, Nanjing, China
Department of Architecture, ETH, Zürich

ER & PARG

Table 1(ER) & 2 (PARG) are used for creating Figure 2.
The results of 1-2,000 realizations are recorded in columns A, B, C.
The results of 2,000-4,000 realizations are recorded in columns E, F, G.
The results of 4,000-6,000 realizations are recorded in columns I, J, K.

The number in i-th row (Column A) denotes the times that the largest component sizes fall into the range [5i, 5(i+1)). Likewise for other columns.
Columns A, E, I: largest component
Columns B, F, J: second largest component
Columns C, G, K: third largest component

PPI network

Table 3 (ER) & 4 (PARG) are used for creating Figure 4.
The results of 1-10,000 realizations are recorded in columns A-E.
The results of 10,000-20,000 realizations are recorded in columns H-L.
The results of 20,000-30,000 realizations are recorded in columns O-S.

i-th row (Column A): the times that the largest component sizes fall into the range [10i, 10(i+1)).
i-th row (Column B): the weighted probability (see Methods section) that the second largest component sizes fall into the range [10i, 10(i+1)).
i-th row (Column C): the weighted probability (see Methods section) that the third largest component sizes fall into the range [10i, 10(i+1)).
i-th row (Column D): the times that the second largest component sizes fall into the range [10i, 10(i+1)).
i-th row (Column E): the times that the third largest component sizes fall into the range [10i, 10(i+1)).
Likewise for columns H-L, O-S.

Tianjin infected cases

Table 5 is used for creating Figure 5.
i-th row: the i-th case of the infected
Column A: case Id.
Column B: age.
Column C: gender. 0: male, 1: female
Column D: date of infection confirmation. Number 1 denotes Feb 01, 2020.
Column E: date of severe symptoms.
Column F: date of recover.
Column G: date of death.
Column H: infected by whom (case Id).
Column I: close contacts.
Column J: where (place Id) get infected.
Column K: home (place Id).
Column L: work place.
Column M: relatives (case Id).
Column N: remarks.

place Id:
-6: the department store (Tianjin)
-5: Tianjin railway (Tianjin)
-3: China
-2: Hubei
-1: Wuhan
The following place ids all refer to districts of Tianjin
0 Xi Qing, 1 He Dong, 2 Nan kai, 3 He Ping, 4 He Bei, 5 Bin Hai, 6 He Xi, 7 Hong Qiao, 8 Ning He, 9 Bao Di, 10 Dong Li, 11 Jin Nan, 12 Bei Chen, 13 Wu Qing, 14 Hei He hospital, 91 town of Lin Ting Kou (belongs to 9 Bao Di).

Tianjin tri
Table 6 (ER) & 7 (PARG) are used for creating Figure 6.
20,000 realizations.
i-th row (Column A): the times that the largest component sizes fall into the range [2i, 2 (i+1)).
i-th row (Column B): the weighted probability (see Methods section) that the second largest component sizes fall into the range [2i, 2(i+1)).
i-th row (Column C): the weighted probability (see Methods section) that the third largest component sizes fall into the range [2i, 2(i+1)).
i-th row (Column D): the times that the second largest component sizes fall into the range [2i, 2(i+1)).
i-th row (Column E): the times that the third largest component sizes fall into the range [2i, 2 (i+1)).

Tianjin duo
Table 8 (ER) & 9 (PARG) are used for creating Figure 7.
20,000 realizations.
i-th row (Column A): the times that the largest component sizes fall into the range [2i, 2 (i+1)).
i-th row (Column B): the weighted probability (see Methods section) that the second largest component sizes fall into the range [2i, 2(i+1)).
i-th row (Column C): the weighted probability (see Methods section) that the third largest component sizes fall into the range [2i, 2(i+1)).
i-th row (Column D): the times that the second largest component sizes fall into the range [2i, 2(i+1)).
i-th row (Column E): the times that the third largest component sizes fall into the range [2i, 2 (i+1)).
